# In vitro and in vivo evidences for innate immune stimulators lactic acid bacterial starters isolated from fermented camel dairy products

**DOI:** 10.1038/s41598-018-31006-3

**Published:** 2018-08-22

**Authors:** Khaled Elbanna, Sahar El Hadad, Abdelrahaman Assaeedi, Alia Aldahlawi, Manal Khider, Alawiah Alhebshi

**Affiliations:** 1 Deptartment of Agricultural Microbiology, Faculty of Agriculture, Fayoum University, Fayoum, Egypt; 2 Department of Biology, Faculty of Applied Science, Umm Al-Qura University, Makkah, Saudi Arabia; 3 Department of Biological Science, Faculty of Science, King Abdulaziz University, Jeddah, Saudi Arabia; 4 ResearchCenter of Genetic Engineering and Bioinformatics, VACSERA, Cairo, Egypt; 5 Immunology Unit, King Fahad for medical research, King Abdulaziz University, Jeddah, Saudi Arabia; 6 Department of Dairy Science, Faculty of Agriculture, Fayoum University, Fayoum, Egypt

**Keywords:** Applied immunology, Applied microbiology

## Abstract

Probiotics are commensals with special characteristics that are essential for the development of the immune system, and may protect mucosal surfaces against pathogens. In this study, a total of 40 lactic acid bacteria (LAB) were isolated from different raw and fermented camel’s milk samples collected from Saudi Arabia (Makkah area) and Egypt (Fayoum), and tested for the probiotic properties. Among them, Pro 4 and Pro 7 isolates exhibited excellent probiotic potential including bile salt (0.2–0.6%), phenol tolerance (0.2–0.4%) and salt tolerance (0.0–10%). Furthermore, both strains exhibited antimicrobial activity against wide range of food-borne pathogens and Dermatophytes with average zone inhibition of 37.5, 35.5, 34.5, 27.5, 25 and 23.5 mm for *Staphylococcus aureus*, *Trichophyton mentagrophytes*, *Escherichia coli*, *Listeria monocytogens*, *Candida albicans* and *Salmonella typhi*, respectively. Furthermore, the *in vivo* study indicated that these strains significantly improved the mucosal immune responses through an increase in expression of TLR2 and IFNγ mRNA in mice intestine as well as increased the synthesis of polyclonal IgG, IgM and IgA in mice blood sera. Accordingly, due to these unique probiotic properties, both selected strains could be potentially used as probiotic starter cultures for fermented dairy foods as well as functional food and health products.

## Introduction

Probiotic bacteria are usually defined as microbial food supplements with beneficial health and therapeutic effects including; improving intestinal tract health; enhancing the immune system, antagonizing the pathogen microorganisms, synthesizing and enhancing the bioavailability of nutrients; reducing symptoms of lactose intolerance and reducing risk of certain cancers^1,2^. According to this definition, an enormous number of bacterial species and genera are considered as probiotics.

Lactic acid bacteria (LAB) are regarded as a major group of probiotic bacteria. This groups of bacteria is Gram- positive, non-spore forming, non-respiring (lack catalase) cocci or rods, acid resistant, bile tolerant and produce antimicrobial substances, including organic acids and hydrogen peroxide and bacteriocins (biologically active protein), nonpathogenic and save to use with the status of General Recognize as Safe (GRAS)^3,4^.

They are commonly used in the production of yogurt, and its fermented products are expected to be functional foods that can contribute to health promotion by regulating immunity in gut-associated lymphoid tissues (GALTs). During the recent years, beneficial therapeutic effects of LAB as a probiotic on the immune response have been extensively studied. The innate immune response serves not only as the first line of defense, but also plays a crucial role in the development of subsequent adaptive immune responses. The majority of evidence from *in vitro* systems, animal models and humans suggest that probiotics can enhance both specific and nonspecific immune responses. These effects are believed to be mediated through activating macrophages, increasing levels of cytokines, increasing natural killer cell activity and/or increasing levels of immunoglobulins^5–7^.

Recently, probiotic genomic and proteomic studies have identified several unique genes and specific secondary metabolic compounds derived from probiotic microorganisms, which mediate immunoregulatory effects. In this context, a set of approximately hundred twenty one genes (about 5–6% of them from *L*. *reuteri* genome) were detected by microarray studies and suggested to contribute to mucosal and systemic immune responses. These genes have been placed in the context of cell signaling and metabolic modeling using bio-informatics approaches including metabolic modeling. Of the probiotic microorganisms studied extensively to date, the lactobacilli have been found most amenable to genetic manipulation and functional analysis of specific genes and operons. Functional genomics have identified systems responsible for bile and acid tolerance, prebiotic transport and metabolism. Furthermore, probiotic bacteria are now being explored as appropriate models for drug/vaccine delivery, due to their closely associated with host immunity and immunomodulatory action^8,9^.

Dairy products are the most common foods which contain probiotic microorganisms. Traditional fermented camel milk is valuable source of food for people living in steppe and arid areas of central Asia. Microflora of the fermented camel’s milk plays the major fermentative role in the acidity, texture, aroma and therapeutic role on improvement of digestion properties and responsible for antimicrobials properties^10–12^. However, the potential probiotics properties of LAB isolated from the traditional fermented camel’s milk and their *in vivo* probiotic effects have rarely been reported. Therefore, this work aimed (i) isolation and screening of lactic acid bacteria with probiotic properties from traditional fermented camel milk from area of Makkah, Saudi Arabia and from Fayoum, Egypt, (ii) phenotypic and genotypic characterization of the most promising probiotic isolates, (iii) evaluation of their potential probiotic properties by feeding the mice, (iv) immunological and physiological studies to measure some innate immune responses characterization includes, detection of TLR gene expressions and detection of some related cytokines and Interferon’s or alteration in any physiological properties.

## Results

### Preliminary screening for lactic acid bacterial strains

A total of 40 LAB were isolated from different raw and fermented camel’s milk samples collected from Saudi Arabia and Egypt based on their antimicrobial activity, tolerance to bile salt and phenol (Table 1). Microscopic investigation and the preliminary characterization showed that, all isolates were Gram positive, non-spore former, non-motile. Among them, 28 isolates were related to lactobacilli shape and 12 isolates were lactococci. The results in Table (1) show that, LAB isolates were varied in their probiotic properties. All LAB isolates exhibited antibacterial activity against *Staphylococcu*s *aureus*, and thirty of them showed activity against *Escherichia coli*, while only twenty-five strains exhibited antimicrobial activity against *Candida albicans*. All LAB isolates exhibited good growth on the MRS medium containing 0.2% bile salt or phenol, and most of them revealed weak growth at 0.4%. Based on the results of *in vitro* probiotic properties tests, the two most promising isolates Pro 4 and Pro 7 were selected for further phenotypic and genotypic characterization, as well as for the *in vivo* immune tests.

**Table 1:** Preliminary screening for probiotic strains isolated from different raw and fermented camel milk.

	Partial characterization of selected isolates									
	Cell shape	Gram stain	Milk clotting	Antimicrobial activity against			Tolerance to bile salt^(*)^ (%)		Tolerance to Phenol^(**)^ (%)	
				*E*. *coli*	*Staph*. *aureus*	*Candida albicans*				
							0.2	0.4	0.2	0.4
Pro 1	Lactobacilli	+	+	32	22	19	+	±	+	±
Pro 2	Lactobacilli	+	+	33	24	18	+	±	+	±
Pro 3	Lactobacilli	+	+	26	20	16	+	+	+	±
Pro 4	Lactobacilli	+	+	37	32	24	+	+	+	+
Pro 5	Lactobacilli	+	+	31	28	20	+	±	+	±
Pro 6	Lactobacilli	+	+	34	27	19	+	+	+	±
Pro 7	Lactobacilli	+	+	40	35	28	+	+	+	+
Pro 8	Lactobacilli	+	+	32	23	−	+	+	±	−
Pro 9	Lactobacilli	+	+	30	24	20	+	+	+	±
Pro 10	Lactobacilli	+	+	29	25	18	+	+	+	±
Pro 11	Lactobacilli	+	+	27	29	19	+	+	+	±
Pro 12	Lactobacilli	+	+	−	26	−	+	+	+	±
Pro 13	Lactobacilli	+	+	32	25	20	+	+	±	−
Pro 14	Lactobacilli	+	+	33	27	17	+	+	+	±
Pro 15	Lactobacilli	+	+	30	29	16	+	+	+	±
Pro 16	Lactobacilli	+	+	27	28	18	+	+	±	−
Pro 17	Lactobacilli	+	+	32	25	−	+	±	+	±
Pro 18	Lactobacilli	+	+	30	20	−	+	±	+	±
Pro 19	Lactobacilli	+	±	−	22	−	+	±	+	±
Pro 20	Lactococci	+	+	29	19	20	+	±	+	±
Pro 21	Lactobacilli	+	+	−	22	−	+	+	+	±
Pro 22	Lactobacilli	+	+	24	24	17	+	+	+	±
Pro 23	Lactobacilli	+	+	−	24	−	+	+	+	±
Pro 24	Lactococci	+	±	30	28	20	+	+	+	±
Pro 25	Lactococci	+	+	29	18	−	+	+	+	±
Pro 26	Lactobacilli	+	+	−	22	−	+	+	±	−
Pro 27	Lactobacilli	+	+	25	24	15	+	−	±	−
Pro 28	Lactococci	+	+	28	21	21	+	±	±	−
Pro 29	Lactococci	+	+	−	27	−	+	±	±	−
Pro 30	Lactococci	+	±	33	24	17	+	±	±	−
Pro 31	Lactobacilli	+	+	−	20	−	+	±	±	−
Pro 32	Lactobacilli	+	+	31	19	18	+	±	+	±
Pro 33	Lactococci	+	+	32	29	18	+	−	±	−
Pro 34	Lactococci	+	+	−	28	−	+	±	±	−
Pro 35	Lactococci	+	+	33	29	17	+	±	±	−
Pro 36	Lactococci	+	+	30	25	20	+	+	+	−
Pro 37	Lactobacilli	+	+	−	17	−	+	±	+	−
Pro 38	Lactobacilli	+	+	23	28	22	+	−	+	−
Pro 39	Lactococci	+	+	−	25	19	+	±	+	±
Pro 40	Lactococci	+	±	32	27	−	+	+	+	±

Notes: Capability of lactic acid bacterial isolates to grow on bile salt or phenol was determine by streaking loopfullof each fresh isolate into MRS agar plates containing (0.2 or 0.4%) phenol or bile salt compared to control (without bile salt and phenol) and anaerobically incubated at 37 °C for 24 h. The Score of growth was recorded as: − negative growth; +/−weak growth; +good growth.

### Phenotypic and genotypic characterization of selected probiotic isolates

**Figure 1:**
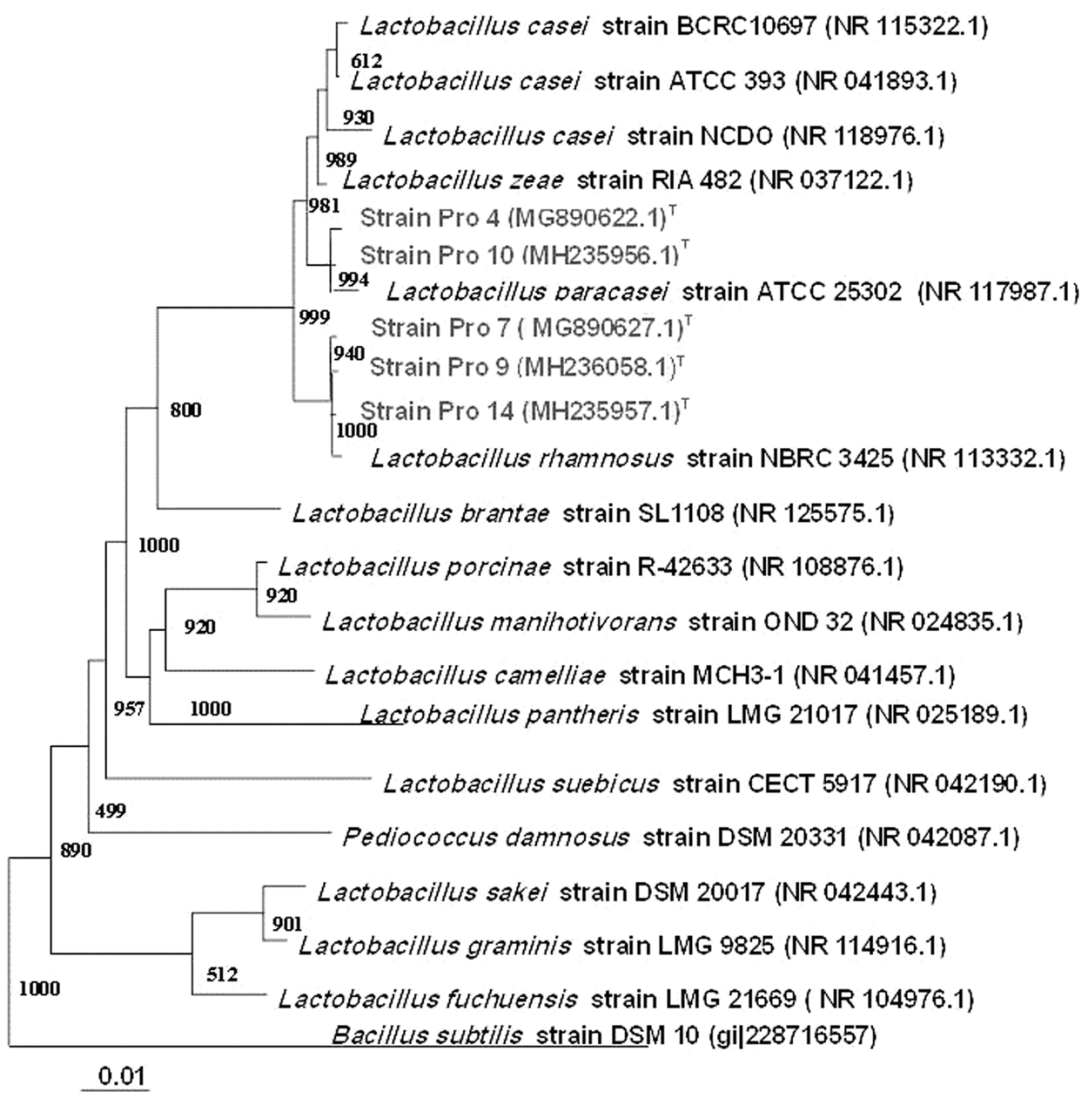
Neighbor-joining tree showing the estimated phylogenetic relationships of the probiotic strains P4 and P7 (shown in gray) and other closely-related strains of the genus *Lactobacillus*. Bootstrap values out of 1000 are given at the nodes. *Bacillus subtilis* strain DSM 10 (gi|228716557) as out group.

Table (2) shows the features that differentiate between the selected isolates (Pro 4 and Pro 7) compared to the reference strains. Both isolates were Gram positive lactobacilli cells, non-spore former, non-motile and could utilize a wide range of carbon sources and differ from the references strains in some features. Both isolates were differing in utilization of L-arabinose, D-xylose, mannose, dulcitol, inositol, arbutin, raffinose, rhamnose, α -methyl-mannoside, amygdalin and gentiobiose. According to the API analytical profile index, the results in Table (2) permitted to identify both isolates to the genera of *Lactobacillus* but could not identify them at the species level. A phylogenetic tree based on 16S rRNA gene sequences (Fig. 1) showed that strains Pro 4 (Accession No. MG890622), Pro 9 (Accession No. MH236058) and Pro 14 (Accession No. MH235957) were similar to *Lactobacillus paracasei* with similarity of 99%, while strain Pro 7 (Accession No. MG890627) and strain Pro10 (Accession No. MH235956) were similar to *Lactobacillus rhamnosus* with similarity of 99%, respectively.

**Table 2:** API 50CHL carbohydrate profile for selected probiotic strains.

	Carbon sources	Pro 4	Pro 7	Reference strains^(*)^	
				*L*. *paracasei*	*L*. *rhamnosus*
1	Glycerol	+++	++	+++	−
2	Erythritol	−	−	−	−
3	D-Arabinose	−	+++	−	+++
4	L-Arabinose	+++	−	+++	++
5	Ribose	+++	+++	+++	++
6	D-Xylose	+++	−	+++	−
7	L-Xylose	−	−	−	−
8	Adonitol	−	−	−	−
9	β-Methyl-D-Xyloside	−	−	−	−
10	Galactose	+++	+++	+++	+++
11	Glucose	+++	+++	+++	+++
12	Fructose	+++	+++	+++	+++
13	Mannose	++	−	+++	−
14	Sorbose	−	−	−	−
15	Rhamnose	−	+++	−	+++
16	Dulcitol	++	−	−	−
17	Inositol	+++	−	−	−
18	Mannitol	+++	+++	+++	+++
19	Sorbitol	+++	+++	+++	+++
20	α-Methyl-D-Mannoside	−	+++	−	+
21	α-Methyl-D-Glucoside	−	−	−	−
22	N-acetyl-glucosamine	+++	+++	+++	+++
23	Amygdalin	−	+++	+++	+++
24	Arbutin	+++	−	+++	+++
25	Esculin Hydrolysis	+++	++	+++	++
26	Salicin	−	−	+++	+++
27	Cellobiose	+++	+++	+++	+++
28	Maltose	+++	+++	+++	+++
29	Lactose	+++	+++	+++	+++
30	Melibiose	+++	+++	+++	+++
31	Sucrose	++	+++	+++	+++
32	Trehalose	+++	−	+++	+++
33	Inulin	−	−	−	−
34	Melezitose	−	+++	−	+++
35	Raffinose	+++	−	+++	+
36	Starch hydrolysis	−	−	−	−
37	Glycogen	−	−	−	−
38	Xylitol	−	−	−	−
39	Gentiobiose	−	+++	+++	+++
40	D-Turanose	+++	+++	+++	+++
41	D-Lyxose	−	−	−	−
42	D-Tagatose	−	−	−	−
43	D-Fucose	−	−	−	−
44	L-Fucose	−	−	−	−
45	D-Arabitol	−	+++	−	+++
46	L-Arabitol	−	−	−	−
47	Gluconate	+++	+++	+++	+++
48	2-Keto-gluconate	−	−	−	−
49	5-Keto-gluconate	−	−	−	−

Notes: Carbohydrate fermentation profiles were applied according to API 50 CHL strips. The Score of the result tests: − negative growth; +weak growth; ++good growth, +++very good growth. (*)Reference strains of *Lactobacillus rhamnosus and Lactobacillus paracasei*obtained from culture collection of department of Agricultural Microbiology and Biotechnology, Faculty of Agriculture, Fayoum University, Egypt.

### Probiotic properties of selected isolates

Probiotics are commensals with special characteristics that are essential for the improvement of the immune system, and may protect mucosal surfaces against pathogens. In the current study, the selected LAB isolates isolated from different raw and fermented camel’s milk were examined for their probiotic properties including the antimicrobial activity, tolerance to different concentrations of bile salt, phenol and NaCl. Out of 40 isolates, the most promising isolates of Pro 4 and Pro 7 exhibited wide spectrum antimicrobial activity against all tested pathogens, as well as good bile salt and phenol tolerance (Table 1).

**Figure 2:**
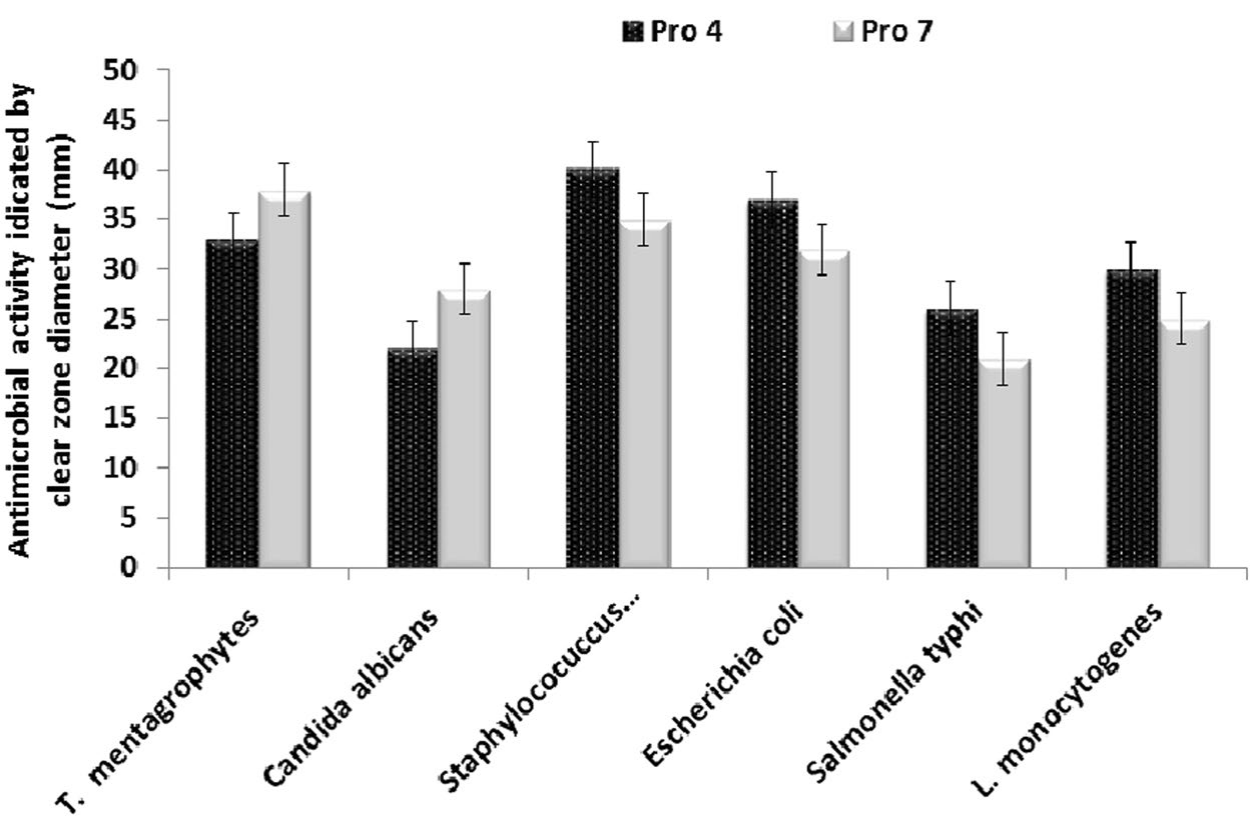
Antimicrobial activity of partial purified bacteriocin of selected probiotic isolates against different food-borne pathogen and Dermatophytes microorganisms indicated by clear zone diameter (mm)^a,b^. ^a^Each value represents mean of sample ± SD for *n* = 3. ^b^Diameter of inhibition zone was measured as the clear area centered on the agar well containing the sample.

Data in Fig. (2) showed that the selected probiotic isolates were differed in their antimicrobial activity against tested pathogens. The highest antimicrobial activity of isolate Pro 4 was recorded against *Staphylococcus aureus* (40 mm), followed by *Escherichia coli* (37 mm), *Trichophyton mentagrophytes* (33 mm), *Listeria monocytogens* (30 mm), *Salmonella typhi* (26 mm) and *Candida albicans* (24 mm), respectively. While The highest antimicrobial activity for isolate Pro 7 was recorded against *Trichophyton mentagrophytes* (38 mm) *Staphylococcus aureus* (35 mm), *Escherichia coli* (32 mm), *Listeria monocytogens* (30 mm) and *Salmonella typhi* (21 mm), respectively. Furthermore, both selected probiotic isolates displayed good resistance to gastrointestinal conditions such bile salt, acid, phenol and NaCl.

**Figure 3:**
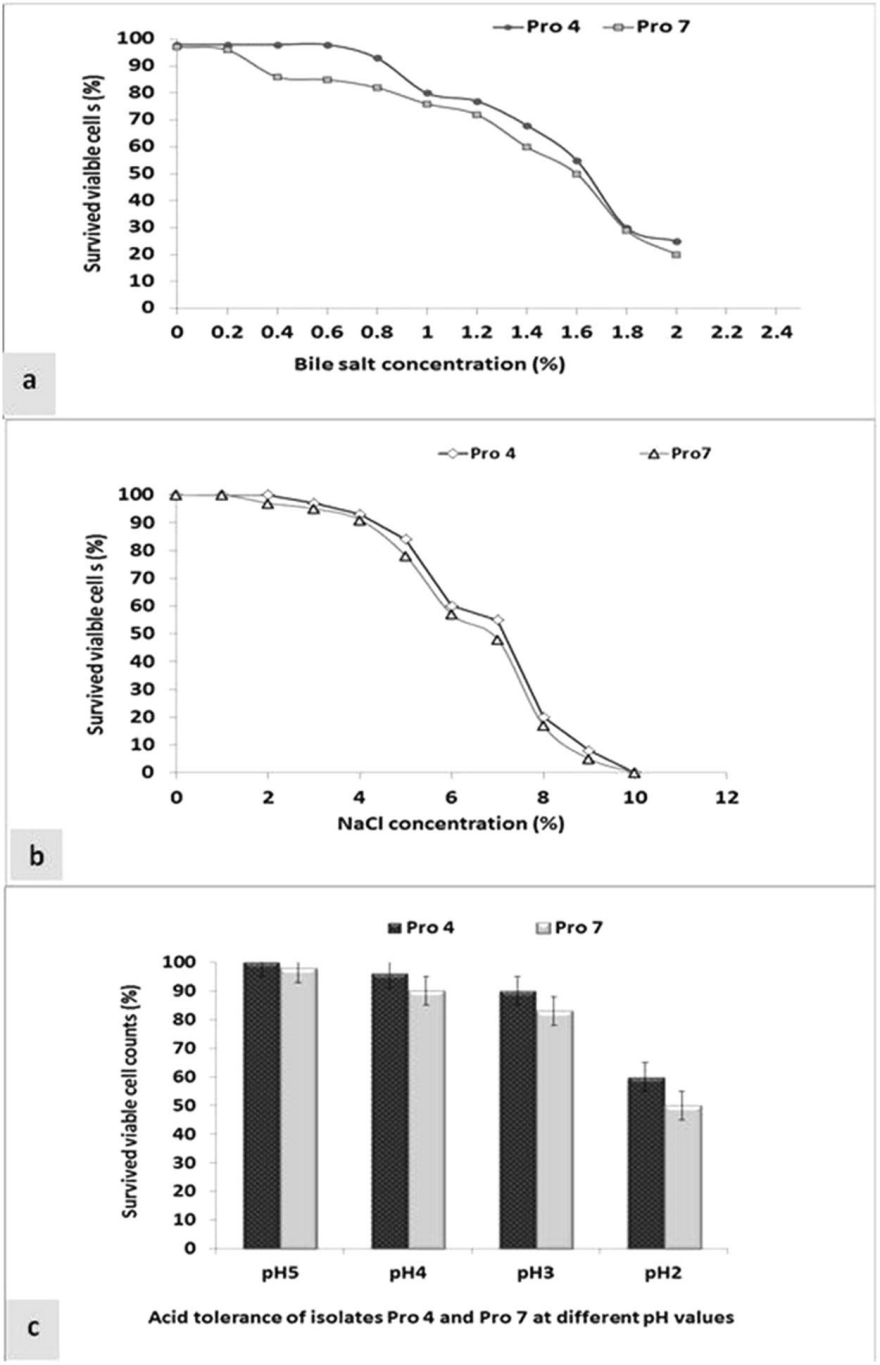
Probiotic properties of selected isolates, bile tolerance (**a**), NaCl tolerance (**b**) and acid tolerance (**c**). Each value represents mean of sample ± SD for *n* = 3.

**Figure 4:**
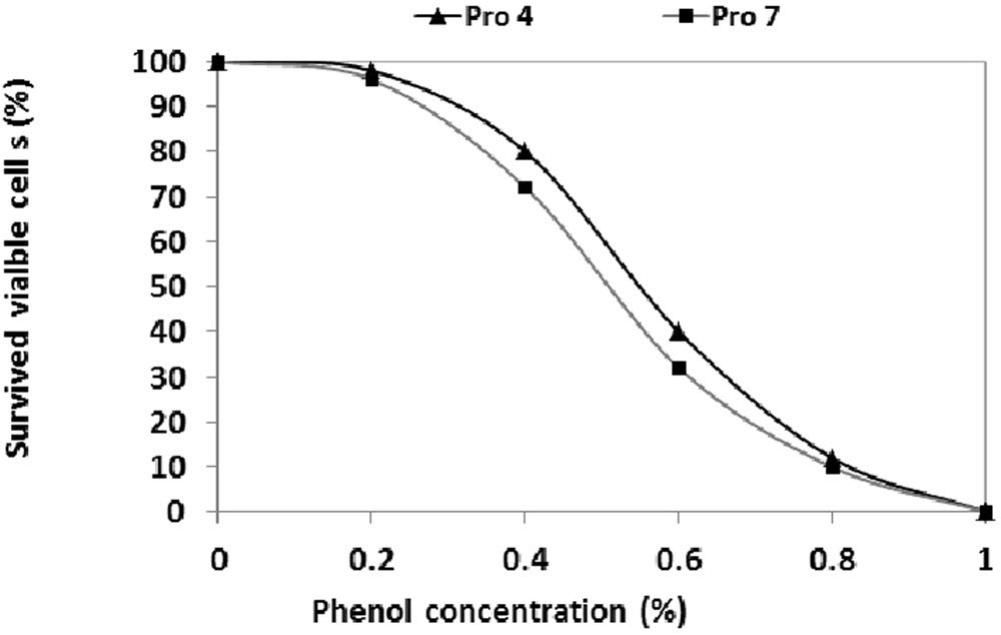
Tolerance of selected probiotic isolates to different phenol concentrations (w/v). Each value represents mean of sample ± SD for *n* = 3.

Results in (Fig. 3a), showed that isolates Pro 4 and Pro 7 exhibited an excellent tolerance to bile salt (0.0–2%) and there is a gradual decline in viable count was observed as the bile concentrations increased. Isolate Pro 4 was more resistance to the bile salt than Pro 7, where it displayed more than 90% survival rate at 0.8%. However, both isolates displayed more than 70% survival rate at 1.0% bile salt, and they kept more than 50% survival rate up to 1.6%. Results in (Fig. 3b) clearly revealed that both isolates were able to tolerate a wide range of NaCl (1–10% w/v) with good growth up to 5% of NaCl, and then the growth was sharply declined with the increase of salt concentration. Both isolates displayed good resistance to low pH (3–5) and drastically decreased at pH 2 (Fig. 3c). Survival rate percentage of isolate Pro 4 was 100, 96, 90 and 60% at pH 5, 4, 3, 2, respectively. While, was 98, 90, 83 and 50% for isolate pro 7 at the same pH values. In addition, both isolates exhibited good tolerance to phenol up to 0.4%, and then the survival rate was drastically decreased as the phenol concentrations increased. Whereas, the survival rates of isolates Pro 4 and Pro 7 were 98, 98, 80 and 72% at 0.2 and 0.4% phenol, respectively (Fig. 4). Moreover, both strains were sensitive to most tested antibiotics (Table 3). They were sensitive to penicillin, clindamycin, oxacillin, erythromycin, gentamicin, tetracycline, amikacin, piperacillin, imipenem and chloramphenicol, while they were resistant to cephalothin, cotrimoxazole, ceftazidime, aztreonam and ciprofloxacin.

**Table 3:** Antibiotic susceptibility of selected probiotic strains.

Antibiotic		Pro 4	Pro 7	Reference strains	
				*L*. *paracasei*	*L*. *rhamnosus*
1	Penicillin G (10 μg)	S	S	R	S
2	Cephalothin (30 μg)	R	R	R	R
3	Clindamycin (2 μg)	S	S	S	S
4	Oxacillin (5 μg)	S	S	R	S
5	Cotrimoxazole (25 μg)	R	R	R	R
6	Erythromycin (15 μg)	S	S	S	S
7	Gentamicin (10 μg)	S	S	S	S
8	Tetracycline (30 μg)	S	S	S	S
9	Amikacin (30 μg)	S	S	S	S
10	Ceftazidime (30 μg)	R	R	R	R
11	Aztreonam (30 μg)	R	R	R	R
12	Piperacillin (100 μg)	S	S	S	S
13	Imipenem (10 μg)	S	S	S	S
14	Ciprofloxacin (5 μg)	R	R	R	R
15	Chloramphenicol (30 μg)	S	S	S	S

Notes: R = resistant, S = Sensitive.

### Regulation of TLR2 gene expression in mice intestine

**Figure 5:**
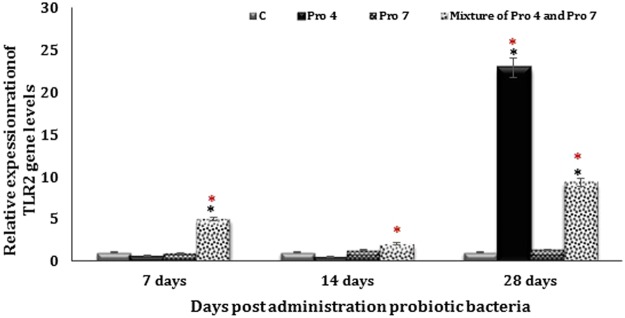
Regulation of TLR2 gene expression in all mice groups following treating with probiotic bacteria. Where *C* represented untreated group Pro 4 represent group treated with *Lactobacillus paracasei/casei*, Pro 7 represent group treated with *Lactobacillus rhamnosus* and mixture of Pro 4 and Pro 7 represent group treated with a mixture of 1:1 from *Lactobacillus paracasei/casei and Lactobacillus rhamnosus*. Total mRNA was isolated from the intestine of the indicated mouse strains and subjected to TLR2 real-time PCR. (*) Significant at p < 0.05 as determined by analysis of variance, comparison was performed using One factor ANOVA test. (*) Comparison between controls and tested group. (*) Comparison between the treated groups. Every point represents the mean value of three separate tests and the vertical bars denote the 5% percentage around the mean.

After 7 days from probiotic treatment, mice group that received Pro 4 strain demonstrated a non-significant down regulation of TLR2 mRNA expression by the mucosal immune cells compared to untreated (C) group (*P* = 0.77). Mice treated with Pro 7 strain seemed more similar to untreated group. TLR2 receptors upregulated significantly in mice group administrated with a mixture of Pro 4 and Pro 7 strains comparing to either Pro 7 and C groups (*P* = 0.0119 and 0.0136) respectively. High significant upregulation (*P* = 0.0078) of the same receptor was observed in mice fed with a mixture of Pro 4 and Pro 7 strains comparing to Pro 4 group (Fig. 5).

Completing analysis after 14 days, the certain receptor level detected in mice intestine biopsy treated with Pro 7 strain still tend to be as similar as the C group, while mice treating with Pro 4 strain demonstrated a non-significant downregulation comparing to either groups received Pro 7 strain (*P* = 0.22) or untreated control (*P* = 0.416). Notably a significant upregulation was observed in the same receptor level extracted from mice treating with the mixture of Pro 4 and Pro 7 strains compared to those treated with Pro 4 strain (*P* = 0.02), and non-significant increase was illustrated compared to either C or Pro 7 groups (*P* = 0.1994 and 0.09) respectively (Fig. 5). No changes were detected in the TLR2 mRNA expression extracted from mucosa of mice intestine treated with Pro 7 strain for 28 days compared to C group. Mice group was given both Pro 4 and Pro 7 mixture strains showed a significant increase in certain receptor expression compared to C and Pro 7 groups (*P* = 0.004 and 0.0051) respectively (Fig. 4). Remarkably, Pro 4 mice group showed a high significant increasing in TLR2-mRNA level compared with either C, Pro 7, or mixture of Pro 4 and Pro 7 groups (*P* = 0.0001, 0.037 and −0.0001) respectively (Fig. 5).

### Regulation of IFNγ gene expression in mice intestine

**Figure 6:**
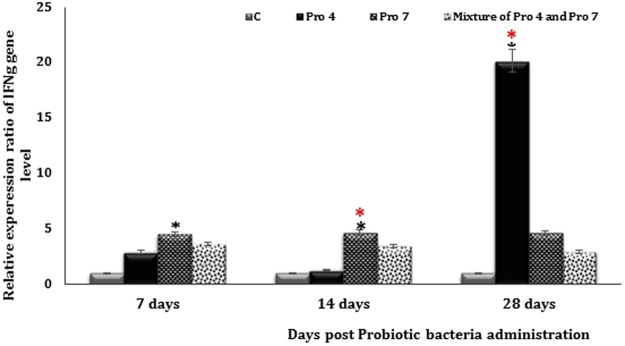
Regulation of IFNγ gene expression in all mice groups following treating with probiotic bacteria. Where *C* represented untreated group Pro 4 represent group treated with *Lactobacillus paracasei/casei*, Pro 7 represent group treated with *Lactobacillus rhamnosus* and a mixture of Pro 4 and Pro 7 represent group treated with a mixture of 1:1 from *Lactobacillus paracasei/casei and Lactobacillus rhamnosus*. Total mRNA was isolated from the intestine of the indicated mouse strains and subjected to IFNγ real-time PCR. (*) Significant at p < 0.05 as determined by analysis of variance, comparison was performed using One factor ANOVA test. (*) Comparison between controls and tested group. (*) Comparison between the treated groups. Every point represents the mean value of three separate tests and the vertical bars denote the 5% percentage around the mean.

To examine the effect of strains Pro 4, Pro 7 and their mixture on Th1-type cytokine expression, mucosal immune cells were stimulated with the present bacteria and expression of IFNγ was measured at 7, 14, and 28 days’ post treatment. Expression of IFNγ-mRNA at 7 days’ post exposure to either Pro 4 isolate, or a mixture of Pro 4 and Pro 7 strains recorded a non-significant increase compared to untreated group (*P* = 0.16, and 0.059) respectively. Meanwhile, IFNγ-RNA expression was increased significantly (*P* = 0.017) by Pro 7 strain post the same time period compared with the C group, while increased non-significantly compared to Pro 4 and mixture of Pro 4 and Pro 7 groups (*P* = 0.567, and 0.508) respectively (Fig. 6).

Mice continuously exposed to Pro 7 strain 3 times weekly for 14 days upregulated significantly the IFNγ-RNA expression compared to C and Pro 4 groups (*P* = 0.003, and 0.007) respectively, while increased non-significantly compared to Pro 4 and Pro 7 group (*P* = 0.259). Otherwise, non-significant increase was detected after the same period in the certain cytokine expression level in mice intestine exposed to either Pro 4, or mixture of Pro 4 and Pro 7 strains compared to C group (Fig. 6).

After 28 days continued providing the current probiotic strains, Pro 4 group showed very high significant increase in the expression of IFNγ-mRNA level compared to C, Pro 7, and mixture of Pro 4 and Pro 7 groups (*P* = 0.0002, 0.0004, and 0.001) respectively. IFNγ-RNA expression levels still increased non-significantly in either Pro 7 strain or a mixture of Pro 4 and Pro 7 groups compared to C group (*P* = 0.33, and 0.606) respectively. Non-significant increase was observed in the same cytokine expression level in Pro 7 group as compared to Pro 4 and a mixture of Pro 4 and Pro 7 groups (*P* = 0.63) (Fig. 6).

### Level of IgG, IgM and IgA in mice blood serum

**Figure 7:**
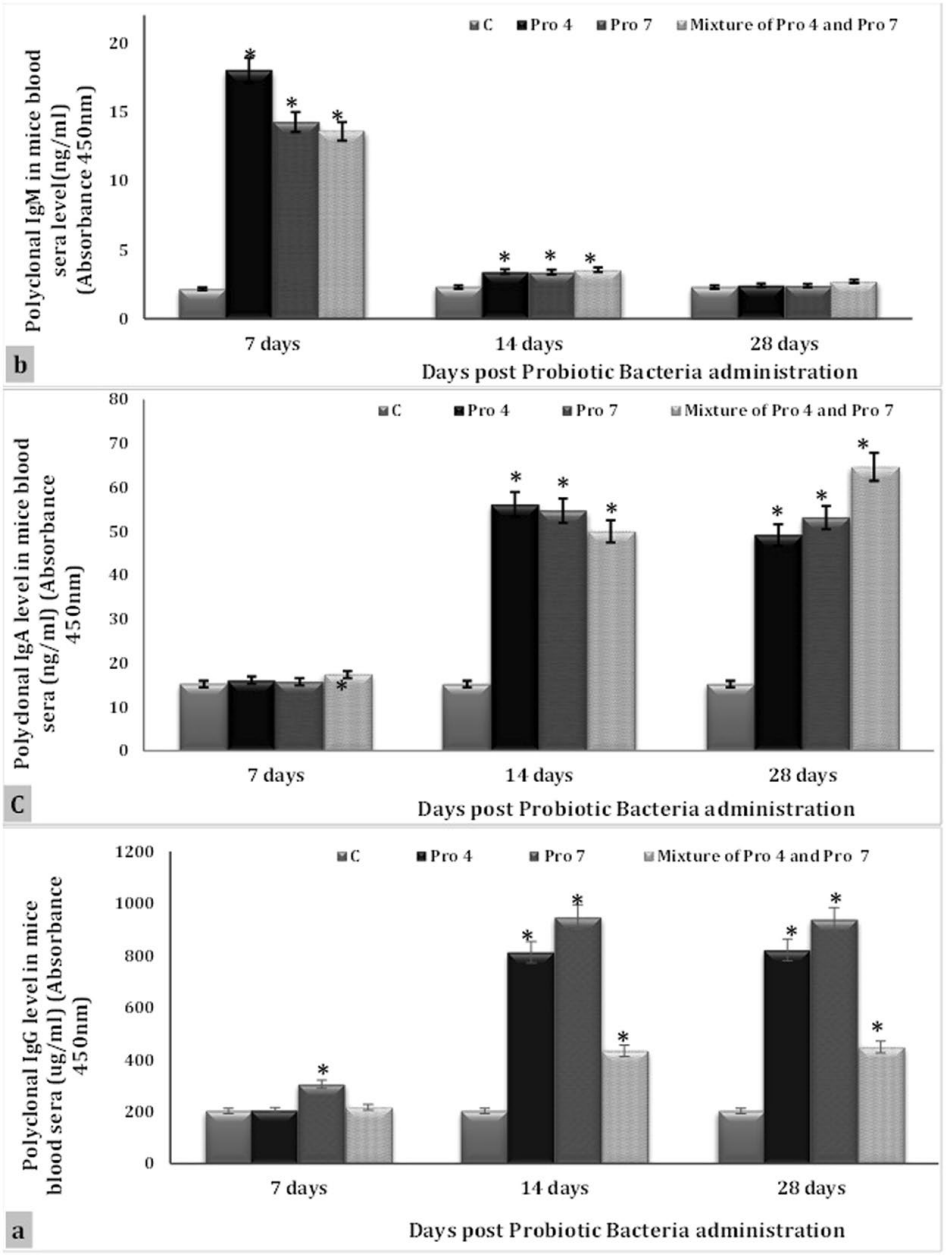
Profile of total serum Immunoglobulins in sera of all mice groups following administration of probiotic bacteria. (**a**) Level of polyclonal IgG, (**b**) Level of polyclonal IgM, and (**c**) Level of polyclonal IgA. Where C represented untreated group, Pro 4 represent *group treated with Lactobacillus paracasei/casei*, Pro 7 represent group treated with *Lactobacillus rhamnosus* and a mixture of Pro 4 and Pro 7 represent group treated with a mixture of 1:1 from *Lactobacillus paracasei/casei and Lactobacillus rhamnosus*. (*) Significant at p < 0.05 as determined by analysis of variance, comparison was performed using One factor ANOVA test. (*) Comparison between controls and tested group. (*) Comparison between the treated groups. Every point represents the mean value of three separate tests and the vertical bars denote the 5% percentage around the mean.

Sera from both untreated and treated mice (strains Pro 4 and Pro 7 or their mixture) were tested to estimate the level of sera polyclonal IgG using capture ELISA. No significant differences were verified in the level of polyclonal IgG between untreated and treated (Pro 4, Pro 7 and their mixture) groups after 7 days, while polyclonal IgG level increased significantly in mice sera treated with Pro 7 strain compared to C, Pro 4, and a mixture Pro 4 and Pro 7 groups (*P* = 0.0005, 0.0006, and 0.00016) (Fig. 7a). An extremely significant increase was detected throughout the day’s 14 (*P* = 0.00007, 0.0001, and 0.0008) and day’s 28 (*P* = 0.000, 0.000, and 0.0001) of period studied in all present treated groups (Pro 4, Pro 7 and mixture of Pro 4 and Pro 7 strains respectively) compared to untreated group. Both Pro 4 and Pro 7 groups illustrated high significant increase in polyclonal IgG as compared to a mixture of Pro 4 and Pro 7 mice group (*P* = 0.0000) in the same time periods. Total IgG increased significantly (*P* = 0.016 and 0.004) in mice sera treated with Pro 7 strain for 14 and 28 days compared to those treated with Pro 4 strain (Fig. 7a).

The current probiotic strains Pro 4, Pro 7 and their mixture increased significantly the circulating total IgM in the mice sera compared to the C group (*P* = 0.000) (*P* = 0.031, 0.04, and 0.025) after 7 days as well as 14 days respectively. Blood sera collected from all treated mice groups post 28 days’ exposure to the current strains seemed more similar to untreated control group (Fig. 7b). Evaluating analysis of the total IgM levels produced post administration of the three current probiotic strains- showed a significant increase in mice obtained Pro 4 strain comparing to Pro 7 and mixture of Pro 4 and Pro 7 mice groups (*P* = 0.0032, and 0.0075) after 7 days from the exposure. In contrast, no significant differences were detected in the certain polyclonal Ig circulating in mice sera of Pro 4, Pro 7 and mixture of Pro 4 and Pro 7 groups after 14 and 28 days (Fig. 7b). With 7 days, polyclonal IgA circulating in mice sear exposed to Pro 4 and Pro 7 strains seemed as similar as to the C group, meanwhile group obtained a mixture of Pro 4 and Pro 7 verified a significant increase compared to C, Pro 4 and Pro 7 mice groups (*P* = 0.0012, 0.008, and 0.0038) respectively (Fig. 7c). All the current treated groups (Pro 4 and Pro 7 and a mixture of Pro 4 and Pro 7 strains) induced a highly significant elevation of circulating total IgA at 14 and 28 days comparing to the untreated group (*P* = 0.000). In addition, high significant increase was observed in polyclonal IgA blood sera of the certain Ig in both Pro 4 or Pro 7 groups compared to group exposed with a mixture of Pro 4 and Pro 7 strains (*P* = 0.0043, and 0.001) after 14 days. In contrasting observation was reported after 28 days where blood sera of mixture of Pro 4 and Pro 7 groups verified a significant increase in the total IgA compared to either Pro 4or Pro 7 groups (*P* = 0.0004 and 0.0025) respectively (Fig. 7c).

## Discussion

In the current study, a total of forty lactic acid bacteria were isolated from different raw and fermented camel’s milk samples collected from Saudi Arabia and Egypt; and were tested for the probiotic properties. Out of them isolates Pro 4 and Pro 7 displayed a wide spectrum antimicrobial activity against both food-borne and Dermatophytes pathogens tested, as well as they exhibited good probiotic properties including; tolerance to bile salt, phenol, high osmotic concentrations of sodium chloride and low pH values (Figs 2–4). Bile salts can influence the intestinal microflora by acting as antimicrobial molecules^13^. However, the average concentration of bile salts in the small intestine is around 0.3%, and it may increase to 2% (w/v), depending upon the type and amount of food ingested^14^. Consequently, when evaluating the potential use of LAB as a probiotic, it is usually important to evaluate their ability to tolerate bile salts. Lee and Salimen, Xanthopoulos *et al*.^15,16^ reported that the ability to tolerate bile salts varies a lot among the LAB species and between strains themselves. Bile resistance of some isolates is related to the enzyme activity of bile salt hydrolase (BSH) that helps to hydrolyze conjugated bile, reducing its toxic effect^17^. Worth mentioning that, tolerance to bile salts has been considered a condition for colonization and metabolic activity of bacteria in the host’s intestine, bile salts can influence the intestinal microflora by acting as antimicrobial molecules. It was reported that, *Lactobacillus* spp. isolates have been shown to be very resistant to low pH, with high survival rates at pH 3.0 for 1 h^13,15^.

Additionally, both isolates Pro 4 and Pro 7 exhibited good tolerance to phenol up to 0.4%, and then the survival rate was drastically decreased as the phenol concentrations increased. These results are in accordance with that reported by Gangadharan *et al*.^18^ who found that *Lactococcus* spp. exhibited high survivability at 0.2% phenol, then decreased to 50% and 10% survival at 0.4 and 0.6% phenol, respectively. Worth mentioning that resistance of the probiotic bacteria to phenol is also an important probiotic factor as some aromatic amino acids are derived from dietary or endogenously produced proteins can be deaminated in the gut by bacteria leading to the formation of phenols. Phenolic compounds can exert bacteriostatic effects, thus testing for resistance to phenol generates information on potential for survival in the gastrointestinal conditions, thereby proving to be the best probiotic strain^19^.

In order to have probiotic effects in the intestinal tract, LAB must be capable of surviving passage through the gastrointestinal tract (GIT). Depending on the specific diet, the pH of the human gastric environment varies from 1.5 to 3.0, and is usually around pH 3^20^ In the present study, isolates Pro 4 and Pro 7 displayed good resistance to low pH (3–5) and drastically decreased at pH 2. Furthermore, both isolates were able to tolerate a wide range of NaCl (1–10% w/v) with good growth up to 5% of NaCl and the growth were sharply declined with the increase of salt concentration. Sodium chloride is an inhibitory substance which may inhibit growth of certain types of bacteria and probiotic organisms have to withstand high salt concentration in human gut^21^. Ibourahema *et al*.^22^ reported that, bacterial cells cultured with a high salt concentration could show a loss of turgor pressure, which would then affect their physiology, enzyme activity, water activity and metabolism. Accordingly, due to these unique probiotic properties, both strains were selected for further identification and to prove their probiotic effects *in vivo* study. According to the API analytical profile index, the results in Table (2) permitted to identify both isolates to the genera of *Lactobacillus* but could not identify them at the species level. Bill *et al*.^23^ indicated that some commercial identification systems often yield good results regarding genus identification but they were not fully adequate at the species level. It was reported that characterization of some *Lactobacillus* to species level by biochemical methods alone is not reliable, because of the considerable variations in biochemical attributes between strains currently considered to belong to the same species^24,25^. However, the taxonomy has changed considerably with the increasing knowledge of the genomic structure and phylogenetic relationships between *Lactobacillus* spp. Generally, when the taxonomy researchers are discriminating between closely-related species of the same genus, 16S rRNA sequencing and DNA-DNA hybridization should be the best methods of choice, in accordance with the proposed molecular definition of species^26^. Detecting and identifying various species of LAB with molecular methods are powerful alternatives to the traditional differentiation of bacteria. In this study, analysis of the partial 16S rDNA sequences placed both isolates of Pro 4 and Pro 7 firmly within the *Lactobacillus* genus, and a comparison to the partial 16S rDNA sequences of other *Lactobacillus* strains in this genus further elucidated their phylogenetic positions (Fig. 1). Generally, two prokaryotic species show differences in 16S rRNA sequence of 3% or more. Based on this genotypic characterization, isolate Pro 4 was clustered with *Lactobacillus paracasei*, while isolate Pro 7 was clustered with *Lactobacillus rhamnosus* with similarity of 99%. The distinctness of Pro 4 from Pro 7 was also supported by their physiological characteristics, such as the ability of Pro 4 to utilize L-arabinose, D-xylose, mannose, dulcitol, inositol, arbutin, and raffinose; and inability to utilize rhamnose, α -methyl-mannoside, amygdalin and gentiobiose. In this context, it was reported that “a bacterial species is generally considered to be a collection of strains that show a high degree of overall similarity and differ considerable from related strain group with respect to many independent characteristics”^27,28^. In addition, one of the most important selection criteria for bacterial strains intended for use in the food industry is concern for their safety^29^. In this regard, the selected probiotic strains Pro 4 and Pro 7 were sensitive to most antibiotics tested and were resistant to some others. These results are in accordance with previously reported data for lactobacilli^30^. Generally, they are sensitive to the Gram-positive spectrum antibiotic erythromycin, the broad-spectrum antibiotics tetracycline and chloramphenicol and the beta-lactam antibiotic ampicillin.

The potential for the positive influence of the gut microbiome through the introduction of probiotics microbes is presently an active area of investigation. Probiotic bacteria, including lactobacilli, are thought to have beneficial effects for the host. Among these benefits, the immunomodulatory activities of these bacteria are of note^5,31^. Pattern recognition receptor (PRRs) are members of non-specific (innate) recognition and it play an important role in molecular basis of microbiota-host interaction which consistent with their location on the mucosal immunity in the intestine and innate immune cells, including macrophages and dendritic cells (DCs)^32^. Toll-like receptors (TLRs) were the first identified PRR which locate on regulatory T cells that exert immunosuppressive activity, and the interference with inflammatory signaling pathways and effector functions^33,34^. TLR-2 recognizes a variety of microbial components such as lipoproteins/lipopeptides from various pathogens, peptidoglycans, and lipoteichoic acid from gram-positive bacteria^35^. The present study verified an early significant upregulation of TLR2-RNA levels extracted from mice intestine biopsy exposed to a mixture of Pro 4 and Pro 7 strains in days 7, 14 and 28 compared to the untreated group, while, group of mice treated with Pro 4 strain established a late significant upregulation in the same RNA receptor (after 28 days) compared to control group. On the other hand, the current Pro 7 bacteria strain fed to mice group didn’t stimulate the TLR2-mRNA expression and tend to be similar to the untreated mice control. Probiotic regulation of mucosal immunity at the gene expression levels in host is both host genetics-dependent and strain specific, person-to-person variation in gene expression was the largest determinant of differences between different *Lactobacillus* strains^36^. Remarkably, various *Lactobacillus* strains induced differential gene-regulatory networks in the human small intestinal mucosa. *L*. *casei* promote the immune balance between Th1–Th2 in mucosal intestine, which organize the regulation of TLR in particular TLR2 and enhance the development of natural killer cells^37^. *Lactobacillus rhamnosus* affected the wound healing by controlling cellular proliferation, and IFN response. *L*. *casei* promote a Th1–Th2 balance in mucosal intestine, which enhances the development of natural killer cells^36^. The present Pro 4 strain stimulated the DC or macrophages and would induce signals to increase the number of TLR-2 receptors individually or as a mixture^38^. The increase in the number of TLR-2-positive cells could indicate an activation of the DC population in intestines Peyer’s patches^39^. However, growing evidence has demonstrated that TLRs were also able to promote adaptive immune responses, mainly indirectly, via DCs. In addition, it was found that the T and B lymphocytes also have PPR, which suggests that PRR signaling could induce/modulate adaptive immune responses directly^40,41^. Also, oral probiotics increased Th1 cytokines while inhibited Th2 cytokines in some atopic children^42^. Similarly, our results showed an increase in the IFNγ-RNA level in mice groups treated with either Pro 4, or mixture of Pro 4 and Pro 7 strains compared to the untreated mice, while the group of mice fed Pro 7 strain bacteria upregulated significantly the expression of IFNγ-RNA compared to the untreated group. These findings proved that continuous administration of the current strains stimulated the proliferation of Th1 cells that affect the expression of IFNγ-mRNA in mice intestine. It was reported that some *Lactobacillus* strains considered appropriate in the context of inflammatory diseases, based on its lower inflammatory potential as reflected by high induction of Th1 responses and IFNγ-mRNA expression^43,44^. The increase of the specific immune response translates into an activation of T and B lymphocytes which causes an increase in the level of circulating antibodies in particularly IgM and IgG. Also, the probiotics influence the production of antibodies mainly IgA in the intestinal lumen^38^. Current research verified an stimulation in the production of total IgG in mice sera collected from Pro 7, Pro 4 or their mixture group compared to the control group showing that probiotic supplementation may influence systemic antibody response, with strains specific effects. These findings are interested science IgG are involved on the immune memory and may contribute to disease prevention in the long term. Corresponding to serum IgM, Pro 4, Pro 7 strains, and their mixture tends to exhibit an early significant increased response in the serum IgM after 7, and 14 days compared to the control group, Regarding serum IgA, a mixture of Pro 4 and Pro 7 strains tended to increase mice serum IgA during the early response (7 days) and during the late response (14, and 28 days) compared to the untreated control; this may be related to the concomitant increase observed in serum IgG^45^. On the contrary, Pro 4 and Pro 7 tended to exhibit a late increased response in serum IgA (day 14 and 28) compared to the control group. In contact with antigens present in the digestive content, the Ig A is very important in the digestive tract, representing a first defense against infection whereas they are secreted into the intestinal lumen as secretor IgA. Also, Ig A can inhibit the adhesion of pathogenic bacteria in the mucosal surface of the digestive tract^38^. Our current result showed that not all the Lactobacillus bacteria were equally effective, where the best performance was found in *Lactobacillus acidophilus* and *casei* (*Lactobacillus GG*). We have shown that *Lactobacillus* strains non-specifically enhance not only the proliferation of B lymphocytes but also antibody synthesis by lymphocytes. In addition, *Bifidobacteria* induced B lymphocytes to be reactive to IL-5, stimulating in increase IgA^42^. The present results revealed that after the period of probiotics use, a significant increase in the levels of specific IgA was observed and also by increasing the concentration of bifidobacterial IgA level was increased earlier. Probiotics can improve the defense function of epithelial cells by the induction of cytokine secretion and the production of immunoglobulins and antimicrobial substances^46^. In agree to our study many previous researches suggested that probiotics induce IgA secretion from plasma cells by increasing the number of IgA producing cells in a strain-dependent manner^47^. In addition, probiotic bacteria are now being explored as suitable models for vaccine/drug delivery, due to their close association with host immunity and immunomodulatory action. Furthermore, recent discoveries are also demonstrating that the roles of probiotic bacteria and the resident microbiota extend far beyond gastrointestinal health^48^.

## Conclusion

In conclusion, as far as we know, this is the first report for isolation and identifying probiotic strains isolated from camel’s fermented milk with excellent probiotic potential including tolerance to high bile salt concentration, low pH, high salt, phenol, and antimicrobial activity against wide range of food-borne pathogens and Dermatophytes. Furthermore, the *in vivo* study indicated that these strains significantly improved the innate immune system. Accordingly, due to these unique probiotic properties, both selected strains could be potentially used as probiotic starter cultures for fermented dairy foods as well as functional food and health products.

## Methods

### Isolation and screening of probiotic bacteria

For the enrichment and screening of probiotic strains, total of thirty raw and traditional fermented camel’s milk samples were collected from Makkah area, kingdom Saudi Arabia and from Fayoum, Egypt. The samples were collected aseptically in sterile bottles kept in an ice-box, and transported to the laboratory. Ten grams of each sample were enriched in 90 mL of the sterilized liquid MRS broth and incubated anaerobically at 37 °C for 48 h in anaerobic incubator (Lab-Line 490 Forced-Air CO_2_ Incubator, Markham, Ontario Canada). 100 μl of each enriched culture were spread onto the surface of de Man, Rogosa and Sharpe (MRS) agar plates (Difco, Sparks, MD, USA). MRS agar Plates were anaerobically incubated at 37 °C for 48 h in anaerobic incubator. Typical colonies of lactic acid bacteria (spindle or punctiform) were picked, purified and partially characterized by API 50 CHL (BioMéríeux, Lyon, France). All isolates were tested for their antimicrobial activity against pathogens microorganisms. The most promising isolates were tested for probiotic potential properties such as antimicrobial activity, tolerance for bile salt, pH and NaCl salt; nontoxicity, non-pathogenicity, ability to modulate immune responses. Based on these properties, isolates Pro 4 and Pro 7 were selected for further phenotypic and genotypic characterizations.

### Phenotypic and genotypic characterization of probiotic isolates

Selected probiotic isolates Pro 4 (Accession No. MG890622) and Pro 7 (Accession No. MG890627) were genotypically characterized based on the sequencing of 16S rDNA gene. For this, genomic DNA from both isolates was isolated according to the method described by Elbanna *et al*.^49^. The 16S rDNA gene was amplified by PCR using universal primers for eubacteria^50^. The sequence of selected isolates and other representatives of *Lactobacillus* strains (retrieved from the NCBI database) were aligned using the computer-program ClustalX^51^. The resulting trees were displayed with Tree View^52^. The phylogenetic reconstruction was done using the neighbour joining method^53^ using *Bacillus subtilis* strain DSM 10 (gi|228716557|) as out group.

### Antimicrobial activity of selected strains

Antimicrobial activity of selected probiotic isolates against the human pathogens including; Gram-positive bacteria (*Staphylococcus aureus* and *Listeria monocytogenes*), Gram-negative bacteria (*Escherichia coli* and *Salmonella enteritidis*), and Dermatophytes (*Trichophyton mentagrophytes* and *Candida albicans*) was assessed according to Khider and Elbanna^54^. For each pathogen strain, Mueller Hinton and potato dextrose (Himedia, Mumbai, India) sterilized agar medium were poured into Petri dishes, then left to solidify at room temperature (25 °C) and swabbed from fresh bacterial or fungal culture strain. Wells of 9 mm diameter in solidified agar were cut using a sterile metal cork borer and filled with 200 µl of partial purified prepared cell free supernatant (20 fold) of each probiotic strain^54^. The inoculated plates were incubated at suitable temperature and time for each pathogen. The antimicrobial activity was determined by measuring the clear zones diameter (CZD) around each well in mm. Distilled water without test compounds was used as a control. All experimental procedures were performed in triplicates.

### Phenol tolerance

To determine the phenol tolerance, 100 µl of overnight grown culture of each isolate was inoculated into 900 µl MRS broth supplemented with different phenol concentration (0.1–0.5%) and incubated at 37 °C for 24 h. Viable cells were counted by plating serial dilutions on MRS agar and incubation anaerobically at 37 °C for 48 h. Phenol tolerance cells were assessed by calculating the ratio of survived viable cell cultured (%) on MRS agar compared to the control (without phenol).

### Tolerance to acidic pH

The selected lactic acid bacterial isolates were cultured overnight in MRS broth at 37 °C. The cell pellet was harvested by centrifugation at 5000 *g* for 5 min and washed twice in sterile saline solution. Serial dilutions of the cells were prepared in phosphate buffer at pH 2, 3, 4 and 5. Samples were incubated at 37 °C for 0, 120, 180 min and viable cells were counted by plating serial dilutions on MRS agar and incubation anaerobically at 37 °C for 48 h. Acid tolerance cells were assessed by calculating the ratio of survived viable cell cultured (%) on MRS agar compared to the initial count (control).

### Tolerance to bile salts

Effect of bile salt on the growth of probiotic strains were tested according to the method of Tsai *et al*.^55^. For this, 9 ml MRS broth with series of oxgall bile salt concentrations (0.2–2%) was prepared in Hungate tube and inoculated with 1 ml of overnight grown culture, Subsequently, incubated at 37 °C for 24 h. The viable cell counts were enumerated by plating 100 µl of each culture onto the MRS agar plates and anaerobically incubated at 37 °C for 48 h. Bile salt tolerance cells were assessed by calculating the ratio of survived viable cell cultured (%) on MRS agar compared control without bile salt.

### Tolerance to sodium chloride salt

To determine the probiotic strain for the salt, MRS broth containing a series of NaCl concentrations (1–10% w/v) was inoculated with overnight grown culture, subsequently, incubated at 37 °C for 24 h. The viable cell counts were enumerated by plating 100 µl of each culture onto the MRS agar plates and anaerobically incubated at 37 °C for 48 h. Salt tolerance cells were assessed by calculating the ratio of survived viable cell cultured (%) on MRS agar compared control without bile salt.

### Hemolytic activity test

The selected strains were examined for blood hemolysis by inoculating MRS agar plates supplemented with 5% sheep blood, followed by incubation at 37 °C for 24 h.

### Antibiotic susceptibility of selected probiotic strains

Susceptibility of the selected probiotic strains to different antibiotics was performed according to EFSA (2012)^29^. MRS agar plates were inoculated by swabbing the LAB fresh cultures onto the surface of agar plates which allowed standing at room temperature for 3 h before antibiotic discs (Mast Diagnostic GmbH, Germany) were applied. Plates were incubated anaerobically at 37 °C for 48 h in the anaerobic incubator. The sensitivity to antibiotics was expressed as sensitive or resistant.

### Milk clotting

To determine the milk clotting by the probiotic strains, each strain was grown overnight on MRS broth, then 1% (v/v) of each culture strain was inoculated into sterilized camel’s milk and incubated at 37 °C for 24 h. Milk coagulation was occurred due to the involvement of lactic acid forming bacteria.

### Preparation of probiotic inoculum

Probiotic strains (Pro 4 and Pro 7) were grown individually in standard MRS culture medium. The inoculated culture medium was incubated at 37 °C for 72 hours and growth of probiotic is recorded at regular intervals. The cultures were centrifuged at 5000 × *g* for 30 min, then the cells pellet was washed twice with saline buffer and resuspended in 50 mM phosphate buffer (pH 6.8) with a final count of 10^7^ CFU/mL and were kept at 4 °C till use.

### Mice and Experiment design

Sixty female BALB/C mice of six to eight-week-old were raised throughout experimentation in the Center of King Fahed for Medical Research at King Abdulaziz University Jeddah, KSA, and maintained under standard laboratory conditions including diet and temperature of 22 °C (+/−2) with continuous supply of water. Its body weights were almost 25–30 g^56^. All mice were divided into 4 groups as following; untreated (C) group included six untreated mice, and they were maintained as a control group. Three groups of fifteen mice were treated three times weekly by gavage with 10^7^ CFU *Lactobacillus paracasei/casei (*Pro 4) group, 10^7^ CFU *Lactobacillus rhamnosus* strain (Pro 7) group, or10^8^ CFU a mixture of 1:1 from *Lactobacillus paracasei/caseiand Lactobacillus rhamnosus strains* (mixture of Pro 4 and Pro 7). All methods and the experimental protocols were performed in accordance with the guidelines for the carefulness and use of Laboratory animals by King Abdulaziz University, Faculty of Science, institutional animal care use committee (IACUC), Saudi Arabia. Five mice from each group (untreated, Pro 4, Pro 7 and mixture) were fasted prior to dissection after 7, 14, and 28 days from the starting of the experiment. They were sacrificed under light ether anesthesia, and blood samples were drawn from the heart for estimation of immunoglobulin level. All mice’s intestine and blood sera samples were stored in −80 °C until used.

### Extraction of mRNA from mice intestine biopsy and Gene expression of TLR2 and IFNγ-mRNA

Mice’ intestines of control group as well as groups treated with probiotic bacteria were subjected to total RNA extraction using RNeasy Maxi kit (QIAGEN, Cat No. 75162) according to manufacturer’s instructions. Pure extracted RNA was eluted using RNase-free water and RNA samples were aliquoted and stored in −80 °C until use.Real time quantitation was performed in Bio-Rad 3600 X instrument (Roche Diagnostics, Laval, Quebec, Canada) using the Verso SYBR Green 1-Step RT-qPCR Master Mix reagents (Thermo Scientific Cat No. AB-4104/C, USA). Previously published primers were used for relative quantification of expression of target genes (encoding TLR2, and IFNγ) and the β-actin gene-actin gene (reference gene), all primers sequences have been mentioned in Table 4^57^. Each PCR reaction (Final volume 20 ul) contained 12.5 ul Verso SYBER Green master mix, 0.25 ul Verso enzyme, 0.25 mM (each) primers, and 400–500 ng RNA samples. cDNA was performed at 50° for 30 min, followed by 40 cycles of 95° 15 sec. 58° 30 sec., and 72° 30 sec. Reference genes Actin transcripts were used for relative quantification. The mRNA relative quantification of target genes was conducted according to the 2-ΔΔCt method. The calibrator sample employed was the same mice sample performed in all runs^58^.

**Table 4:** Primers used for TLR2, IFNγ, andβActin genes expression quantitation using SybergreenqRT-PCR.

Gene	Polarity	Primer sequence (5′-3′)	Primer length	Nucleotide positions^(*)^	Accession numbers of References genes
β Actin	Sense	ATGGATGATGATATCGCCGCG	21	547–566	NM001101
	Antisense	CTAGAAGCA1TfGCGGTGGACGATGGAGGGGCC	32	746–725	
TLR2	Sense	AACCTCAGACAAAGCGTCAAATC	22	323–345	NM_011905
	Antisense	ACCAAGATCCAGAAGAGCCAAA	21	387–366	
IFNγ	Sense	GGCCATCAGCAACAACATAAGCGT	22	364–387	NM_008337
	Antisense	TGGGTTGTTGACCTCAAACTTGGC	22	481–458	

(*)Nucleotide position numbering is based on reference genes sequences that were retrieved from DDBJ/EMBL/GenBank.

### Assay of total IgA, IgM, and IgG in mice blood sera using Capture ELISA

Plasma IgA, IgM, and IgG concentrations were determined in appropriately diluted samples by a sandwich ELISA using pre-coated microtiter plates and mouse specific IgA, IgM, and IgG ELISA quantitation kits (Abcame, United Kingdom, Cat No, ab157719, ab133047, ab157717) respectively. The ELISA procedure was carried out according to the protocol of the manufacturer and absorbance was measured at 450 nm. The concentrations of IgA, IgM, and IgG were determined using standard curves constructed from respective Ig standards run on the assay microtiter plate and were expressed as milligrams of IgA, IgM, or IgG per milliliter of plasma. The enzyme reaction with the substrate yields a colored product. Color intensity is proportional to the amount of specific antibody and total plasma Ig concentration was calculated as the sum of the respective plasma IgA, IgM, and IgG concentration.

### Statistical Methods

Analysis was performed using Megastat software, and the one-way ANOVA parametric test was conducted to determine the statistical significance between the untreated group as well as groups treated with Pro 4, Pro 7, and a mixture of Pro 4 and Pro 7, where appropriate p-value of <0.05 was considered significant.
