# Publisher Correction: In vitro and in vivo evidences for innate immune stimulators lactic acid bacterial starters isolated from fermented camel dairy products

**DOI:** 10.1038/s41598-019-42891-7

**Published:** 2019-06-18

**Authors:** Khaled Elbanna, Sahar El Hadad, Abdelrahaman Assaeedi, Alia Aldahlawi, Manal Khider, Alawiah Alhebshi

**Affiliations:** 1 Deptartment of Agricultural Microbiology, Faculty of Agriculture, Fayoum University, Fayoum, Egypt; 2 Department of Biology, Faculty of Applied Science, Umm Al-Qura University, Makkah, Saudi Arabia; 3 Department of Biological Science, Faculty of Science, King Abdulaziz University, Jeddah, Saudi Arabia; 4 ResearchCenter of Genetic Engineering and Bioinformatics, VACSERA, Cairo, Egypt; 5 Immunology Unit, King Fahad for medical research, King Abdulaziz University, Jeddah, Saudi Arabia; 6 Department of Dairy Science, Faculty of Agriculture, Fayoum University, Fayoum, Egypt

Correction to: *Scientific Reports* 10.1038/s41598-018-31006-3, published online 22 August 2018

The original version of this Article contained errors.

Reference 58 was inadvertently omitted.

In addition, References 33 to 57 were incorrectly cited in the Article.

These errors have now been corrected in the HTML and PDF versions of this Article.

